# Editorial: Placental Hormones and Pregnancy-Related Endocrine Disorders

**DOI:** 10.3389/fendo.2022.905829

**Published:** 2022-04-28

**Authors:** Qiongjie Zhou, Ganesh Acharya

**Affiliations:** ^1^ Department of Obstetrics, Obstetrics and Gynecology Hospital of Fudan University, Shanghai, China; ^2^ Department of Clinical Sciences, Intervention and Technology, Karolinska Institute, Stockholm, Sweden; ^3^ Center for Fetal Medicine, Karolinska University Hospital, Stockholm, Sweden; ^4^ Department of Clinical Medicine, Faculty of Health Sciences, UiT - The Arctic University of Norway, Tromsø, Norway

**Keywords:** Placental Hormones, Pregnanacy, Endocrine disorders, Environmental factor, Preeclampsia, Gestational diabetes, Hypothyroidism

Metabolic changes occur continuously in the mother and her fetus during pregnancy, and the role of hormones in maintaining normal fetal growth and development cannot be overemphasized. The scale of endocrine control needed to support physiological functions during pregnancy is clearly demonstrated by the drastic changes in hormone profiles and their fluctuations observed from preconception to the postnatal period. Differential sensitivity to the fluctuation of placental hormones can also play a role in the development of perinatal mental health disorders. Thus, endocrine homeostasis is important for a successful pregnancy and its favorable outcome. Placenta is an important endocrine organ in this regard as it plays a crucial role in mediating maternal physiological adaptation to pregnancy.

Endocrine disorders during pregnancy have become a major concern in recent years due to their rapidly increasing prevalence (1, 2) as well as their adverse impact on maternal and offspring health (3). Placental dysfunction remains a root cause of many pregnancy-associated endocrine disorders. This could be associated with several intrinsic as well as extrinsic factors, including genetic aberrations as well as functional changes related to dietary habits, lifestyle factors and environmental exposures.

With emerging new technologies in molecular biology, together with the real possibility of using mega-data and artificial intelligence, significant progress has been made in our understanding of key regulatory mechanisms involved in the pathophysiology of pregnancy related endocrine disorders, associated risk factors, and their short- and long-term consequences. The topic of “Perinatal Endocrinology and Pregnancy-related Endocrine Disorders” in Frontiers in Endocrinology presents 14 high quality original research and review articles on this theme contributed by researchers from around the world. The focus is both on basic science exploring pathophysiological mechanisms as well as clinical practice of screening, diagnosis and management of pregnancy-associated endocrine disorders.

Regarding physiological endocrine changes in pregnancy, Garces et al. focus on angiopoietin-like protein 3 (ANGPTL3) that plays a significant role in lipoprotein metabolism. They measured its levels in healthy nonpregnant women, pregnant women at different trimesters of gestation and in the postpartum period. They report lower levels of ANGPTL3 during pregnancy which may promote lipid uptake by highly oxidative tissues leading to preservation of glucose for the growth and development of the feto-placental unit during pregnancy.

Three papers address the pathophysiological characteristics of pregnancy-associated endocrine disorders. Cathey et al. have addressed an interesting topic exploring the association between gestational hormones and timing of delivery. In this longitudinal cohort study, higher corticotropin releasing hormone (CRH), estriol, progesterone, total triiodothyronine and free thyroxine were observed among male fetuses with spontaneous preterm birth and higher testosterone in female fetuses, indicating intrauterine sexual dimorphism in the association between hormones and preterm birth. Han et al. have investigated the variations in adiponectin, leptin, insulin and ghrelin levels in the umbilical cord blood of preterm infants and further elucidated their link with fetal growth. A lower concentration of these hormones was found in preterm and small for gestational age infants, suggesting an impaired maturation of adipose tissue and gastrointestinal tract. The authors hypothesize that dysregulation of these hormones may be a risk factor for abnormal fetal growth. Liu et al. have explored the associations between triglycerides, lymphocyte subsets and insulin resistance among patients with polycystic ovarian syndrome. Interestingly, elevated level of triglycerides was significantly associated with higher risk of hyperinsulinemia and impaired immune response among patients with insulin resistance and recurrent pregnancy loss, providing a new insights into the pathophysiology of these conditions.

Two studies have reported on regulatory molecular mechanisms underlying uterine receptivity, decidualization of endometrium, and placentation. Ashour et al. have used a rat model of vitamin D deficiency to investigate the role of HOXA-10/FKBP52 axis in regulating uterine receptivity. Downregulated HOXA-10/FKBP52 was noted in vitamin D-deficient rats, in association with increased amplitude and frequency of uterine contractility. More importantly, they show that vitamin D supplementation might be potentially useful for improving endometrial decidualization, uterine receptivity, and reducing myometrial contractility. On the other hand, Feng et al. demonstrate the role of collagen I in suppressing the proliferation and invasion capacity of trophoblasts by inhibiting ERK phosphorylation and WNT/b-catenin signaling pathways to induce preeclampsia-like symptoms.

Our Research Topic also discusses the new concepts in the pathogenesis and regulatory mechanism of thyroid disease in pregnancy and postpartum period. Synthesizing currently available evidence based on high-quality randomized studies, Girolamo et al. have concluded that levothyroxine (LT4) supplementation is not effective in improving adverse pregnancy outcomes among euthyroid pregnant women with thyroperoxidase (TPO) antibodies. Gao et al. (4), have evaluated maternal postpartum thyroid function within one year after delivery among women with pre-existing or newly diagnosed hypothyroidism in early pregnancy. They found that women diagnosed to have subclinical hypothyroidism in early pregnancy were at higher risk requiring LT4 treatment within one-year postpartum if they stopped LT4 treatment at delivery, had abnormal TSH level before eight gestational weeks and near delivery, or had TPOAb≥300 mIU/mL. Thus, a modified strategy for close monitoring of thyroid function during early pregnancy and postpartum period may be indicated. Additionally, Kankanamalage et al. have written a broad review on the pathogenesis of gestational hypothyroidism. The concept of gestational hypothyroidism as a separate entity of thyroid hormone dysregulation during pregnancy is proposed. Possible mechanisms could be impaired iodine regulation, elevated estrogen levels, malplacentation and dysregulation of placental endocrine function.

Studies on pregnancy-related metabolic disorders, especially gestational diabetes, and their impact on pregnancy outcome have also been included in this themed issue. Wang et al. have explored the association between increased fibroblast growth factor 21 (FGF21) and subsequent development of gestational diabetes mellitus (GDM), utilizing a nested case-control design. The authors report that serum FGF21 level is significantly elevated several weeks before the diagnosis of GDM is made, suggesting a potential value of FGF21 in predicting GDM. Bai et al. have utilized the GDM model to investigate the role of angiopoietin like 8 (ANGPTL8), a secretory protein involved in lipid metabolism, in GDM and insulin resistance. They have demonstrated that the silencing of ANGPTL8 promotes insulin resistance in trophoblast cells by inhibiting JNK signaling. Han Z. et al. assessed the changes of placental volume and vascular indices in pregnant women with GDM using three-dimensional power Doppler, and found them to be significantly lower in the GDM group in the first trimester compared to healthy pregnant women. This noninvasive approach might be useful in assessing the degree of placental vasculopathy in GDM. In addition, Han S. et al. conducted a retrospective cohort study to investigate the risk of adverse pregnancy outcomes among women with diminished ovarian reserve. An increased risk of hypertensive disorders of pregnancy and slightly elevated incidence of preterm birth were observed.

The role of environmental factors on placental development and function was also addressed in this Research Topic. Per- and poly-fluoroalkyl substances (PFAS) are persistent pollutants, which accumulate in humans *via* food, drinking water, outdoor air, etc (5, 6). Marchese et al. assessed the gestational characteristics of placental brain-derived neurotrophic factor (BDNF) signaling and the impact of PFAS exposure on this pathway in trophoblast. They report that BDNF signaling is diverse during placental development and is affected by PFNA exposure. This PFAS potentially interferes with the key signaling pathway of BDNF in fetal neurodevelopment.

We believe that this Research Topic has been enriched by the inclusion of basic and clinical studies and will appeal to both clinicians and scientists equally. We hope that it will improve our understanding of pregnancy-related endocrine disorders, and motivate further research into perinatal endocrinology and pregnancy associated endocrine disorders.

## Author Contributions

Both authors conceptualized and wrote the manuscript. All authors contributed to the article and approved the submitted version.

## Funding

QZ was funded by the National Key Research and Development Program (2021YFC2701600, 2021YFC2701601), Clinical Research Plan of SHDC (SHDC2020CR1047B), the Shanghai Excellent Young Scholar Plan of Public Health (2020-2022, GWV-10.2-YQ13), Elite Young Scholar 2025 of Fudan University (2020-2023), Shanghai Medical Center of Key Programs for Female Reproductive Diseases (2017ZZ01016), and the Science Foundation of Shanghai (21ZR1410600). The funders had no role in study design, data collection and analysis, decision to publish, or preparation of the manuscript.

## Conflict of Interest

The authors declare that the research was conducted in the absence of any commercial or financial relationships that could be construed as a potential conflict of interest.

## Publisher’s Note

All claims expressed in this article are solely those of the authors and do not necessarily represent those of their affiliated organizations, or those of the publisher, the editors and the reviewers. Any product that may be evaluated in this article, or claim that may be made by its manufacturer, is not guaranteed or endorsed by the publisher.
